# Punctate inner choroidopathy and optic neuropathy: simultaneous presentation in a patient - a case report

**DOI:** 10.1186/1869-5760-3-54

**Published:** 2013-06-27

**Authors:** Michelle V Carle, David S Boyer, Pouya N Dayani

**Affiliations:** 1 Retina Vitreous Associates Medical Group, 1127 Wilshire Blvd, Ste 1620, Los Angeles, CA 90017, USA

**Keywords:** Multifocal choroiditis, Punctate inner choroidopathy, Optic neuropathy, Mycophenolate mofetil

## Abstract

**Background:**

We present a case of a patient initially presenting with multifocal choroiditis (MFC) in one eye. She subsequently developed lesions most consistent with punctate inner choroidopathy (PIC) in the contralateral eye, followed by acute vision loss from retrobulbar optic neuropathy. Optic neuropathy has been well described in the setting of MFC. There is, however, only one report of its association with PIC. Punctate inner choroidopathy and MFC have many similarities, with visual loss generally resulting from choroidal neovascularization. In this case, the patient had significant visual loss from presumed retrobulbar optic neuropathy.

**Findings:**

The patient responded well to immunomodulation with subsequent return of vision to baseline.

**Conclusions:**

Multifocal choroiditis and punctate inner choroidopathy may be a spectrum of the same disease with many overlapping presentations, including optic neuropathy. Good visual recovery and remission were attained with mycophenolate mofetil and systemic corticosteroid treatment.

## Findings

This report describes a case of recalcitrant posterior uveitis with characteristics of both multifocal choroiditis (MFC) and punctate inner choroidopathy (PIC), which was subsequently complicated by optic neuropathy in the eye with lesions most consistent with PIC.

Optic neuropathy appears to be more commonly associated with MFC than PIC [1,2]. Optic neuropathy in patients with MFC has been well described as presenting with optic disc edema or pallor, with a typical response to steroids resulting in an increase in visual performance [2]. A review of the literature identified only one case report of the association of PIC and optic neuropathy [1].

There remains controversy whether PIC and MFC (and perhaps other white dot syndromes) represent similar disease processes on a continuum [3,4]. The present case describes a patient presenting with lesions characteristic of multifocal choroiditis in one eye and punctate inner choroidopathy in the contralateral eye. Optic neuropathy was the primary cause of visual loss in the eye with lesions more typical of those seen in PIC. The presentation of optic neuropathy (ON), more commonly seen with MFC, demonstrated in the eye with uveitic characteristics of PIC, further unifies these disorders. In addition to reporting this rare association, this case highlights the overlap of PIC and MFC by demonstrating gradual progression and enlargement of the chorioretinal lesions, which were initially more typical of those seen in PIC.

### Case report

**Figure 1:**
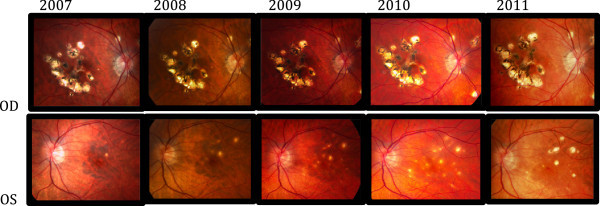
**Macular Lesions 2007-2011.** Initial evolution of chorioretinal lesions over a 4-year period to disease activity (fourth frame OS) then quiescent on treatment in 2010: disease stability in 2007, 2008, 2009, 2010 (active disease), and 2011 (quiescent disease). Notice that OD remains stable throughout, and OS remains stable until a considerable increase of activity of lesions occurs in the 2010 photos but returns to quiescent lesions in 2011.

**Figure 2:**
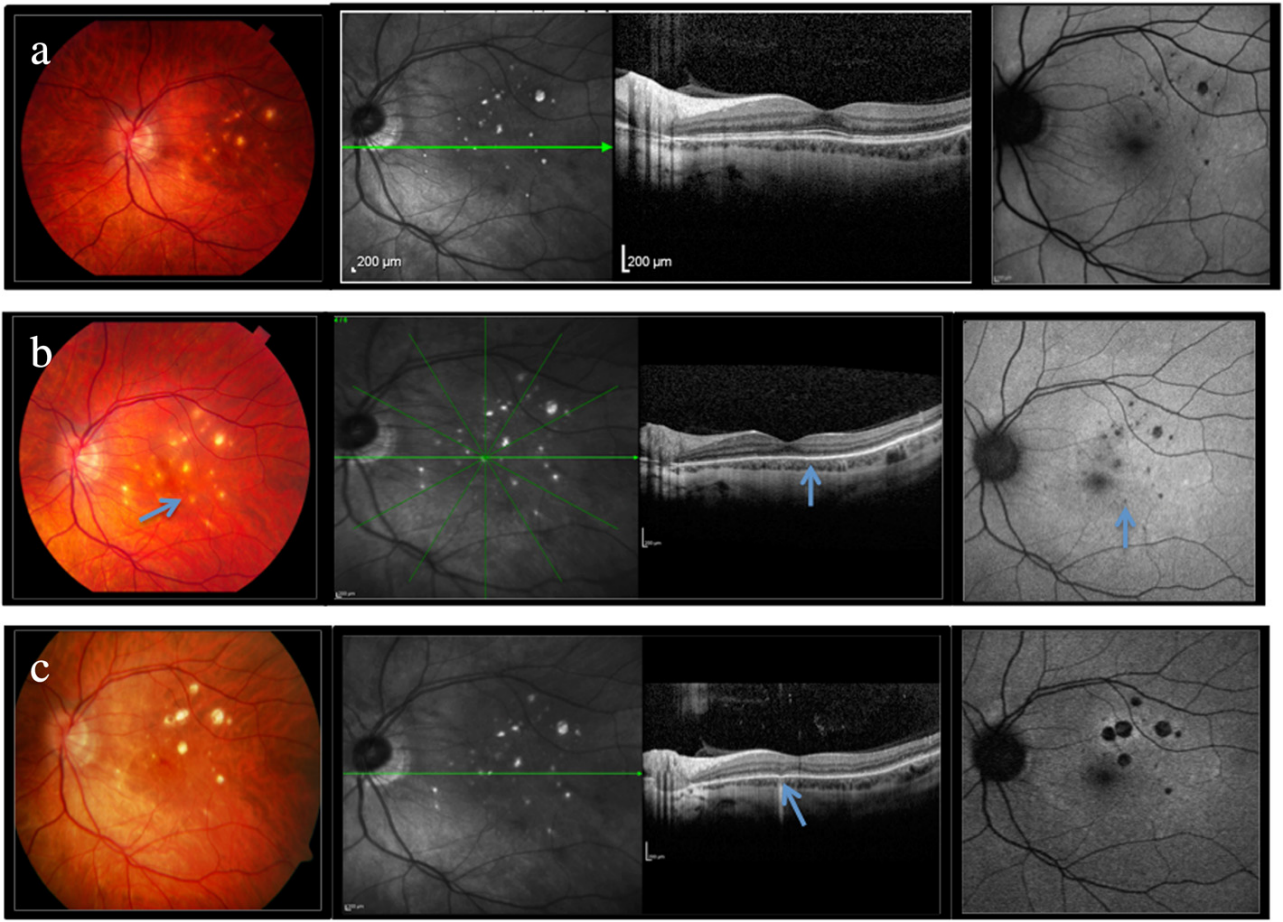
**Comparative evolution of PIC lesions OS.(a)** Fundus and red-free photograph, spectral domain optical coherence tomography (SD-OCT), and autofluorescence at baseline of our report (early 2010). **(b)** Active disease with multiple new lesions in the left eye 3 months later when the vision have fallento CF. Note the new lesions (regions indicated by arrows) on color photograph and autofluorescent image, and retinal pigment epithelium (RPE)/photoreceptor alterations (arrow) on SD-OCT image during active disease. No optic nerve edema or hyperemia is noted. **(c)** Most recent images following treatment demonstrating regression of some of the smaller lesions and enlargement of others. SD-OCT image showing RPE/photoreceptor loss in the areas of previously active lesions (arrow).

The patient is a 30-year-old myopic woman (approximate refractive error of −7.50 diopter) with a history of recurrent macular and midperipheral chorioretinal lesions typical of MFC (without vitritis) for 5 years in the right eye (see Figure 1 - OD (*oculus dexter*), 2007 to 2011). She had been treated with oral and periocular steroids for exacerbations. After several years, the inflammatory process in the right eye underwent a quiescent phase without the development of any further lesions. At that time, the best-corrected visual acuity (BCVA) was 20/50 in the right eye and 20/20 in the left eye. Over the ensuing 3 years, recurrent inflammation was observed in the left eye only (see Figure 1 - OS (*oculus sinister*), 2007 to 2009). The lesions in the left eye were smaller and more typical of those seen in PIC (see Figure 1 - OS and Figure 2a). Although nasal and midperipheral lesions were seen in the right eye, only macular lesions were observed in the left eye. A typical systemic evaluation was performed and was negative for any specific inflammatory or infectious process (including tuberculosis, Lyme, syphilis, Bartonella, sarcoid, and human leukocyte antigen (HLA) A-29 and B-27).

In 2010, the patient presented with vision changes for 3 days in her left eye and reduced visual acuity to 20/25. No cellular inflammation was noted. Dilated posterior segment examination showed an increase in macular chorioretinal lesions. Despite a strong recommendation for systemic immunosuppressive therapy, the patient opted for local treatment, and a posterior subtenon's triamcinolone injection (40 mg) was administered.

Three days later, the patient returned with progressive ‘blurred vision and not much pain’. The visual acuity was reduced to 20/80, but the fundus examination was unchanged. An intravitreal injection of Triesense (2 mg) was administered, and the patient was started on oral prednisone of 80 mg daily and mycophenolate mofetil 1 g BID. The rheumatology service was consulted.

Eleven days later, the patient returned with BCVA of counting fingers in her left eye. The anterior chamber was quiet in both eyes, and the anterior vitreous showed 0.5+ cells in the affected eye. Dilated ophthalmoscopy did not reveal any change from the prior visits. No optic disc edema or pallor was noted (see Figure 2b). The patient had a fluorescein allergy which prevented the acquisition of fluorescein images. Indocyanine green angiography was obtained, which demonstrated lesions corresponding to those seen on clinical examination, optical coherence tomography, and fundus autofluorescence. There were no new chorioretinal lesions or choroidal neovascularization. The patient was pharmacologically dilated, preventing an evaluation for an afferent papillary defect.

Extensive ancillary testing was performed including: a Goldman visual field which revealed an enlarged blind-spot in the left eye; absent color vision in the left eye (unable to see test plate); full field electroretinogram (ERG, normal in both eyes); multifocal ERG (abnormal left eye greater than right eye); and visual evoked potentials (VEP) (normal in the right eye, very low amplitude with normal implicit times in the left eye). The series of testing implied an optic nerve conduction deficit for the left eye.

The patient was admitted to the hospital for intravenous solumedrol (1 g daily for 3 days) and was evaluated by rheumatology, neurology, and neuroophthalmology. The visual loss remained stable with a slight discomfort to the eye during her admission. Magnetic resonance images of brain and spine, with and without contrast, and a lumbar puncture were unremarkable. Images of the optic nerve from the brain MRI did not demonstrate any abnormality. Additional laboratory testing was performed (including extensive rheumatologic and coagulopathic studies), which provided no evidence of a systemic or local etiology for the optic neuropathy. The patient was discharged from the hospital on mycophenolate mofetil and oral steroids taper.

Follow-up examination 1 month after discharge demonstrated that the BCVA had improved to 20/40 in the left eye but detection of color plates remained absent. The visual acuity continued to improve over the subsequent 2 months to 20/25 without any improvement in color vision.

One year following the initial episode of optic neuropathy, the patient's uveitis was stable on mycophenolate 500 mg twice daily. BCVA remained stable at 20/60 for the right eye and 20/20 for the left eye, and color plates were now full for both eyes. Fundus examination shows enlargement of the previous chorioretinal lesions (see Figure 2c).

## Discussion

Punctate inner choroidopathy is a bilateral uveitic disease, primarily affecting young myopic women first described by Watzke in 1984 [5,6]. The primary etiology of this inflammatory condition remains unclear, but an association with Epstein-Barr virus and with HLA-DR2 positivity have been reported [7,8]. The disease typically presents with yellow-white chorioretinal lesions at the level of the choroid, not associated with vitritis. As the lesions evolve, they leave a depigmented halo and appear ‘punched out’ similar to lesions observed in ocular histoplasmosis syndrome (OHS).

Multifocal choroiditis is also a bilateral condition typically affecting myopic women that presents with punched out posterior pole and peripheral chorioretinal scars with juxtapapillary scarring (also similar to OHS) [4]. It was first described by Nozik and Dorsch, and later by Dreyer and Gass, and can be associated with panuveitis and snowbanking, choroidal neovascularization, and visual field defects [9-11]. MFC is not associated with any HLA specificity and is often associated with vitreous and anterior chamber cells.

Many features of this case are typical of both MFC and PIC. Central visual loss in both PIC and MFC, when present, is usually from subretinal fibrosis secondary to choroidal neovascular membranes [5,11,12]. The aspect of this case that is most unique is the sudden severe visual loss without evidence of choroidal neovascularization, foveal chorioretinal lesions, or cystoid macular edema. The initial increase in the chorioretinal lesions and the subtle decrease in visual acuity (to 20/25) were consistent with active inflammatory disease observed clinically. The sudden further loss of central vision (to count fingers), the loss of color vision, the low amplitude of the VEP in the affected eye, and the absence of any new macular lesions led us to believe that the dramatic vision loss was a result of an anterior optic pathway process independent of the lesion activity.

The use of mycophenolate has been shown to reduce the proliferation of B and T lymphocytes through inhibition of purine biosynthesis and can be efficacious in the management of ocular inflammation, including PIC [13,14]. When local therapy became insufficient in the management of our patient, initiation of systemic immunomodulation was effective in controlling the inflammation and maintaining long-term quiescence.

Limitations of this retrospective study are that an afferent pupillary defect was not noted as either being present or absent and the MRI study did not specifically review the optic nerves. The presumed retrobulbar optic neuropathy, however, did demonstrate a typical course and response to treatment. Retrobulbar ON in the setting of PIC has been reported just once by Yamada et al. [1]. This complication, however, is well established in the setting of MFC and has been shown to be steroid responsive [1,2].

The co-presentation of posterior segment lesions characteristic of both PIC and MFC, as well as the subsequent development of optic neuropathy in this patient, is unique. These observations further support the possibility that PIC and MFC are variable presentations of the same disease process, as opposed to discrete clinical entities. The patient initially responded well to local therapy but eventually required a steroid-sparing agent for long-term inflammatory control.

## Abbreviations

MFC: Multifocal choroiditis; PIC: Punctate inner choroidopathy; ON: Optic neuropathy; BCVA: Best-corrected visual acuity; ERG: Electroretinogram; VEP: Visual evoked potentials; HLA: Human leukocyte antigen; OHS: Ocular histoplasmosis syndrome.

## Competing interests

The authors declare that they have no competing interests.

## Authors’ contributions

MC drafted the manuscript. DB provided clinical consultation for the patient and editing of the manuscript. PD provided clinical care for the patient and editing of the manuscript. All authors read and approved the final manuscript.
